# Ulnar and Median Fascicular Transfers for Elbow Flexion-Predicting Outcomes in a Heterogeneous Patient Group and Implications for Surgical Planning

**DOI:** 10.3389/fsurg.2020.567602

**Published:** 2020-12-04

**Authors:** Scott Ferris, William Alexander

**Affiliations:** ^1^Department of Plastic, Hand and Faciomaxillary Surgery, Alfred Health, Melbourne, VIC, Australia; ^2^Victorian Plastic Surgery Unit, St. Vincent's Private Hospital, East Melbourne, VIC, Australia; ^3^Department of Plastic and Maxillofacial Surgery Unit, Royal Children's Hospital Melbourne, Parkville, VIC, Australia; ^4^Victorian Hand Surgery Associates, Fitzroy, VIC, Australia

**Keywords:** brachial, plexus, elbow, nerve, transfer, Oberlin

## Abstract

**Purpose:** To measure the outcomes in patients undergoing nerve transfers for elbow flexion restoration, and compare patient outcomes based on the pre-operative fascicular transfer plan.

**Methods:** Single surgeon series of 48 consecutive patients who underwent median and/or ulnar fascicular nerve transfers for elbow flexion restoration to treat palsies of the brachial plexus or musculocutaneous nerve. Outcomes measured were Medical Research Council (MRC) power grade, strength in kilograms, and time taken to recover function.

**Results:** Overall, 96% of patients achieved MRC M4 or greater power. The subgroup who were planned for, and particularly those who then underwent, double as opposed to single fascicular transfer, had significantly better results.

**Conclusions:** Overall results were excellent. Double fascicular transfers were superior, with no failures in this group. If pre-operatively a single fascicle transfer alone is planned due to a paucity of expendable donors, the predicted outcomes are worse and other treatment options should be considered.

## Introduction

Brachial plexus injuries are catastrophic events that predominantly affect the working-age male population, resulting in partial or complete motor and sensory loss in the upper limb. Re-establishing elbow flexion is a major goal of reconstruction in upper plexus injuries (1, 2).

Primary nerve repair, tendon transfers, and nerve grafts have all been utilized in brachial plexus surgery, but each have significant individual limitations (3–12). Nerve transfer offers a unique solution, allowing reconstruction away from the zone of injury, and a donor source of axons close to the target muscle. This means that regenerating axons only need to cross a single coaptation, with a shorter distance to target, thereby reducing the extent of motor endplate degeneration whilst awaiting reinnervation (12).

In 1994, Oberlin et al. described the transfer of a single, expendable ulnar nerve fascicle to the biceps branch of the musculocutaneous nerve (13). In a subsequent series of 32 patients, he reported successful [British Medical Resource Council (MRC) grades M3 or M4] re-establishment of elbow flexion in 24/32 cases (14). Similar results were reported by subsequent authors- Loy et al. (15), Leechavengvongs et al. (16), and Sungpet et al. (17). The original authors describe poorer results particularly in patients with C5-6-7 injuries (as opposed to C5-6 only), the elderly, and in those with greater delay to operation (18). Despite this, Socolovsky et al. found nerve transfers to be twice as likely as nerve grafting to return M3 or better elbow flexion (19); and it was found to be the most successful nerve procedure (of transfer or grafting) aimed at restoring elbow flexion, in a systematic review (20).

It has been argued that the biceps muscle is primarily a forearm supinator, and a secondary elbow flexor; in contrast to the brachialis, which is the primary elbow flexor (12, 21–23). Regardless of these muscles' specific individual contribution, it would seem logical that innervating two, as opposed to one, muscles across a joint would increase power.

Mackinnon et al. further refined this concept and in 2005 published the “Double Fascicular Transfer” (DFT). This involves the transfer of a median nerve fascicle to brachialis in addition to the ulnar fascicle to biceps. They reported excellent results in six patients (22). One year later in 2006, Liverneaux et al. also reported success in ten patients treated with this technique (18), and these results have been validated by subsequent publications (24). Both early authors, and others since, agree that double fascicle transfers result in superior results (18, 22, 25–28). Carlsen et al. retrospectively compared their results from single and double nerve fascicle transfers, and highlight that those patients receiving single nerve transfers (SFT) had significantly worse pre-operative function (21). Those with more severe injury involving impaired median nerve function are unable to undergo a double fascicle transfer, as the extra donor fascicle required is not available. The authors do not identify which patients had a SFT but were planned to have a DFT, and this particular group of patients is highlighted in our study. We believe that this group is significantly different to those patients who are planned to have a SFT pre-operatively, and warranted specific investigation and description.

The objective of this study was to evaluate the outcomes of what we observed to be a historically heterogeneous group, undergoing similar but different nerve transfers using median and ulnar fascicular donors for elbow flexion reconstruction. In particular, we compared four distinct groups of patients based on their preoperative surgical intent as well as ultimate surgical procedure. The first group underwent planned double fascicular transfers (DFT, group 1). The second group were planned for double fascicular transfers, but an intra-operative decision was made to perform a single fascicle transfer only (unplanned single fascicular transfer, USFT, group 2). The third group underwent planned single fascicular transfers for completely deficient elbow flexion, when preoperative evaluation demonstrated that double fascicular transfers would not be safely possible = (PSFT-M0, group 3). The fourth group underwent planned single fascicular transfers for augmentation of some already existing elbow flexion, when double fascicular transfers were deemed unnecessary (PSFT-Aug, group 4). We defined “double fascicular nerve transfer” as two targets being innervated (brachialis and biceps), regardless of where the donor nerve fascicles originated.

## Materials and Methods

After obtaining institutional ethics board approval, we reviewed our prospectively maintained brachial plexus database. We included all consecutive patients who underwent a single or double fascicular nerve transfer procedure for elbow flexion over an 8-year period. The four groups of patients (DFT, USFT, PSFT-M0, and PSFT-Aug) were compared with respect to demographics, general injury/disease details, plexus injury level, and post-operative function measured in terms of strength (MRC grade and kilograms lifted), and time to best power outcome. Forty-eight patients were identified who underwent nerve transfer for elbow flexion.

Strength was graded using the British Medical Research Council's system. Grade 5 strength was only awarded if it was equivalent to the contralateral arm on clinical resisted manual muscle testing. Strength was also quantified in kilograms lifted to the nearest 500 g. The patient was asked to stand upright with their back straight and hand fully dependent, and actively flex the elbow whilst holding a weight in their hand. Ninety degrees of elbow flexion was the minimum required to record a quantitative lift result, and the heaviest weight possible under these criteria was recorded.

Data were further analyzed in terms of severity of injury. We grouped our patients similarly to Carlsen et al. (21) into the following categories: those with peripheral nerve injuries (<C5–6 group), those with isolated upper trunk injuries (C5–6), and those with more severe injury, extending beyond the upper trunk (>C5–6).

### Surgical Technique

**Figure 1:**
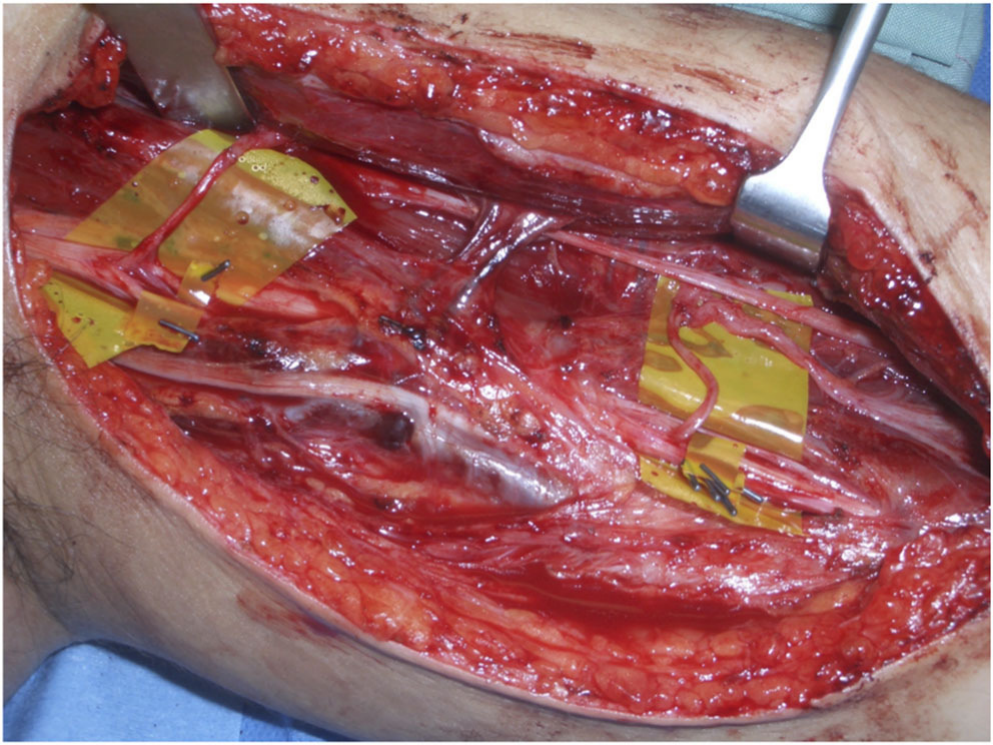
Typical surgical layout, showing fascicular dissection and transfer.

The senior author (SF) performed all operations. Through a medial proximal arm incision the median, ulnar and musculocutaneous nerves were dissected. All individual branches to biceps and brachialis muscles were neurolysed and labeled. An intraneural neurolysis was undertaken as required of the ulnar and median nerves, using the operating microscope. Each individual fascicle once dissected, was labeled, insulated, and selectively stimulated, to inform the decision as to which fascicles were sufficiently powerful and redundant to be used for nerve transfer. This information was tabulated to make decision making easier. After this individual fascicular mapping and decision making, neurotomies were performed on the donor fascicles, followed by transferring the donors to recipient biceps and/or brachialis nerves for secondary nerve repairs (Figure 1). 9–0 monofilament nylon was used for nerve repair under the operating microscope in all cases. Fibrin glue was applied to the outside of the completed nerve repair. In general ulnar fascicles were transferred to biceps and median fascicles to brachialis, although in some instances this was reversed, especially in the single fascicular transfer group (see **Tables 5**, **6**). In the vast majority of single fascicular transfers, biceps was the target of the nerve transfer. There were some cases in which brachialis was the only nerve transfer target, and in all these cases, the reason was that there was already significant function in biceps.

Nerve repairs were tension free through full elbow range of motion and patients were kept in a shoulder immobilizing sling with the elbow at 90 degrees for 2 weeks.

### Statistical Analysis

The data was analyzed in terms of means and medians (depending on nature of data) for continuous variables, and counts and percentages for categorical variables. The focus of the analysis was comparing the functional outcomes between the different patient groups.

## Results

Forty-eight patients were included in the study, and demographic details are shown in Table 1. The majority of the patients were working age males, and the predominant causative mechanism was motor vehicle accident. Almost half of the cohort had a plexus injury involving more than C5 and C6 (44%). The average delay to operation was 5 months, and average follow-up was 21 months post-operatively. Eighty five percent of the cohort had M0 strength pre-operatively, and 96% achieved M4 or better post-operatively, with an average quantitative lift of 5.8 kg. The average time between the operation and patients then first achieving M4 was 9 months.

**Table 1:** Patient demographics.

**Focus**	**Variable**	**Total**	**DFT**	**USFT**	**PSFT-M0**	**PSFT-Aug**	***p*-value**
Total *N*		48	36 (75%)	5 (10%)	3 (6%)	4 (8%)	
Sex	Female	3 (6%)	3 (8%)	0 (0%)	0 (0%)	0 (0%)	1.00
	Male	45 (94%)	33 (92%)	5 (100%)	3 (100%)	4 (100%)	
Age (yrs)	Median (IQR)	33.0 (25.0, 48.0)	32.0 (25.5, 50.0)	25.0 (22.0, 34.0)	48.0 (31.0, 48.0)	42.5 (30.0, 48.5)	0.45
Diagnosis	<C5, 6	14 (29%)	7 (19%)	2 (40%)	1 (33%)	4 (100%)	0.014
	C5, 6	13 (27%)	13 (36%)	0 (0%)	0 (0%)	0 (0%)	
	>C5, 6	21(44%)	16 (44%)	3 (60%)	2 (67%)	0 (0%)	
Delay to operation (m)	Median (IQR)	5.0 (3.5, 7.0)	4.0 (3.0, 5.5)	5.0 (4.0, 9.0)	6.0 (5.0, 7.0)	12.5 (9.0, 17.0)	0.008
Other injuries		37 (77%)	27 (75%)	6 (100%)	3 (100%)	2 (50%)	0.31
Follow-Up (m)	Median (IQR)	21.5 (15.0, 32.0)	25.0 (16.0, 32.0)	21.0 (14.0, 24.0)	19.0 (15.0, 22.0)	17.5 (13.0, 40.5)	0.74

The majority of patients were in the DFT group (36), with five patients in the USFT groups, four patients in the PSFT-Aug group, and three in the PSFT-M0 cohort. The age and sex demographics were similar between the groups. High-speed motor vehicle accidents accounted for the majority of cases (34/48), along with 6 surgical complications, 1 tumor resection, 1 gunshot wound, 1 neuritis, and 5 non-vehicle related traumatic accidents. The distribution of these injuries was similar amongst the patient groups. The majority of our patients (37/48, 77%) had significant concomitant injuries (including long bone, pelvic, or spinal fractures, closed/open brain injury, pneumothorax, or significant vascular injury). The distribution of these was similar across treatment groups.

The severity of plexus injury was significantly different between the treatment groups. Less extensive plexus injuries (<C5, 6) were much more common in the PSFT-Aug group. Conversely, those who were intended to undergo double fascicular transfer, regardless of what procedure actually took place, had more extensive palsies (mostly > C5, 6). The PSFT-M0 group also had more extensive plexus injuries.

The delay to treatment was longer in the PSFT-Aug group (median 12.5 months), when compared to the DFT, USFT, and PSFT-M0) groups (4.0, 5.0, and 6.0 months, respectively).

Medical Research Council elbow flexion grade data was available for forty-six patients, and the 2 patients with missing data were in the DFT group. The two patients who were lost to follow up were excluded from the analysis. Overall, forty-four out of forty-six patients achieved at least M4 power, by 9 months post-operatively, on average. There were significantly improved outcomes in the DFT, USFT, and PSFT-Aug groups when compared to the PSFT-M0 groups (Table 2). The PSFT-Aug patients all had M4 power pre-operatively, and the PSFT-M0 group had the only two patients with poor post-operative results in our study population.

**Table 2:** Operative outcomes.

**Focus**	**Variable**	**Total**	**DFT**	**USFT**	**PSFT-M0**	**PFST-Aug**	***p*-value**
Total *N*		48	36 (75%)	5 (10%)	3 (6%)	4 (8%)	
Pre-Operative MRC	0	41 (85%)	34 (94%)	4 (80%)	3 (100%)	0 (0%)	0.001
	3	1 (2%)	0 (0%)	0 (0%)	0 (0%)	1 (25%)	
	4	6 (13%)	2 (6%)	1 (17%)	0 (0%)	3 (75%)	
Post-operative MRC (*n* = 46)	1	1 (2%)	0 (0%)	0 (0%)	1 (33%)	0 (0%)	0.021
	2	1 (2%)	0 (0%)	0 (0%)	1 (33%)	0 (0%)	
	3	0 (0%)	0 (0%)	0 (0%)	0 (0%)	0 (0%)	
	4	23 (50%)	16 (47%)	4 (80%)	1 (33%)	2 (50%)	
	5	21 (46%)	18 (53%)	1 (20%)	0 (0%)	2 (50%)	
Outcome (kg)	Median (IQR)	5.8 (3.8, 8.3)	6.5 (5.0, 8.5)	4.3 (1.3, 8.5)	0.0 (0.0, 2.0)	6.0 (3.0, 14.0)	0.055
Time to MRC 4 (m)	Median (IQR)	9.0 (6.0, 13.0)	8.5 (6.0, 13.0)	16.0 (10.0, 25.0)	11.0 (11.0, 11.0)	6.0 (6.0, 6.0)	0.35

Quantitative strength data was available for thirty-six post-operative patients. This data was unavailable for earlier patients, as we only began this specific quantitative strength measurement part way through the study period. It is now collected both pre- and post-operatively in our institutions, for all patients. The median post-operative elbow flexion quantitative strength was 5.8 kg. The DFT and PSFT-Aug groups had greater median weight lifts than the USFT and PSFT-M0 groups (6.8 and 6 kg vs. 4.3 and 0 kg, respectively). Pre-operative lift recordings were available for three of the four PSFT-Aug patients, and these patients improved from 4 to 8 kg, 4 to 10 kg, and 0 to 2 kg respectively. The patient without a pre-operative measurement was able to lift 20 kg post-operatively.

In order to compare technique (rather than intention) outcomes, and to align with the published literature, we compared single vs. double fascicular transfers regardless of the pre-operative intention [DFT vs. SFT (USFT + PSFT-Aug + PSFT-M0)] (Table 3). We acknowledge that this comparison is contrived in our groups, because with the exception of the augmentation group, the surgeons' preference whenever safely possible, was to undertake a DFT. SFT was only undertaken for either paucity of donors or for augmentation. As a result the injuries in the SFT group were either >C5, 6 or <C5, 6, but no patients in this group had pure C5, 6 injuries. In contrast to this, pure C5, 6 injuries accounted for more than a third of the patients in the DFT group (Table 4). The DFT group exhibited a greater gain in strength measured by MRC grade, and a trend toward greater final quantitative strength, measured in kilograms, compared to the SFT group.

**Table 3:** Double vs. single fascicle transfer, regardless of intention.

**Focus**	**Variable**	**DFT**	**SFT**	***p*-value**
Total *N*		36 (75%)	12 (25%)	
Sex	Female	3 (8%)	0 (0%)	0.56
	Male	33 (92%)	12 (100%)	
Age (yrs)	Median (IQR)	32.0 (25.5, 50.5)	38.0 (23.5, 45.5)	0.67
Diagnosis	<C5, 6	7 (19%)	7 (58%)	0.008
	C5, 6	13 (36%)	0 (0%)	
	>C5, 6	16 (44%)	5 (42%)	
Delay to operation (m)	Median (IQR)	4.0 (3.0, 5.5)	7.5 (5.0, 9.5)	0.003
Pre-operative MRC	0	34 (94%)	7 (58%)	0.007
	3	0 (0%)	1 (8%)	
	4	2 (6%)	4 (33%)	
Post-operative MRC	1	0 (0%)	1 (8%)	0.04
	2	0 (0%)	1 (8%)	
	3	0 (0%)	0 (0%)	
	4	16 (46%)	7 (58%)	
	5	16 (46%)	3 (25%)	
Outcome (kg)	Median (IQR)	6.5 (5.0, 8.5)	2.0 (1.0, 8.0)	0.085
Time to reach MRC 4 (m)	Median (IQR)	8.5 (6.0, 13.0)	11.0 (9.0, 21.0)	0.29

**Table 4:** Outcomes based on level of plexus injury.

**Focus**	**Variable**	**<C5, 6**	**C5, 6**	**>C5, 6**	***p*-value**
Total *N*		14 (29%)	13 (27%)	21 (44%)	
Pre-operative MRC	0	7 (50%)	13 (100%)	21 (100%)	<0.001
	3	1 (7%)	0 (0%)	0 (0%)	
	4	6 (43%)	0 (0%)	0 (0%)	
Post-operative MRC	1	0 (0%)	0 (0%)	1 (5%)	0.020
	2	1 (8%)	0 (0%)	0 (0%)	
	3	0 (0%)	0 (0%)	0 (0%)	
	4	3 (23%)	5 (42%)	15 (71%)	
	5	9 (69%)	7 (58%)	5 (24%)	
Outcome (kg)	Median (IQR)	7.5 (4.8, 8.8)	5.0 (5.0, 10.0)	4.0 (2.0, 7.0)	0.20
Time to reach MRC 4 (m)	Median (IQR)	6.0 (6.0, 8.0)	6.0 (5.5, 10.5)	11.5 (11.0, 20.0)	0.005

In our population neither an age >30 years old, nor a delay to theatre >6 months, had a statistically significant effect on the primary outcome measures.

## Discussion

This study presents a detailed comparison between patients undergoing single or double fascicular nerve transfers for the restoration of elbow function.

The major findings of this study are that overall outcomes are excellent with 96% (44/46 patients) achieving a M4 or M5 power. Historically, M3 has been considered a success after nerve palsy reconstruction, but in the modern era of nerve transfer surgery we believe the minimum outcome that can be considered a functional success when restoring elbow flexion is not M3, but M4. 100% of patients in this study planned to undergo DFT achieved a post-operative result of M4 or M5, whether or not they ultimately proceeded to DFT or in fact underwent USFT.

The findings presented here are unique in that they highlight several sub-groups of patients who underwent single fascicle transfer, some of which have not been described in any detail previously in the literature. These patients may all have undergone single fascicular transfer, but they are far from a homogeneous group, with different surgical indications and different outcomes. Firstly, there are the single fascicular nerve transfer patients who were, in fact, preoperatively intended for a double fascicular nerve transfer. This USFT group made up a small but significant (10%, 5/48) part of our cohort. Secondly there are those patients with some existing but weak elbow flexion, for whom it was deemed that a single fascicular transfer could realistically augment peak power (PSFT-Aug) to maximize function. Thirdly this study identifies and separately evaluates those patients who underwent planned single fascicular transfers, due to a known paucity of donor availability (PSFT-M0). The clinical reasoning for the operative decisions made in the single fascicle transfer cases (both planned and unplanned) have been described and are summarized in Tables 5, 6.

**Table 5:** Clinical details and operative reasoning for planned single fascicle transfers.

**Age**	**Delay to surgery (m)**	**MOI**	**Palsy**	**Other injuries**	**Surgical details**	**Reason for SFT**	**Pre-Op MRC**	**Post-Op MRC**	**Post-op KG**	**Follow Up (m)**
***PSFT-M0***										
48	7	MBA	MCN + Med	Humeral #	Ulnar n fascicles × 2 to Biceps. Graft from proximal med. N. to directly neurotise brachialis	Median n not functioning.	0	2	0	15
31	6	MBA	C5,6,7+	ABI	Med n fascicles x 2 to biceps	Extensive plexus injury—extreme paucity of donors	0	1	0	19
48	5	Fell off roof	Upper trunk, posterior cord	Nil	Med n fascicle to biceps	Proximal injury to ulnar nerve hence unavailable for transfer	0	4	2	22
***PSFT-Aug***										
54	10	MVA	n. to Biceps	Central cord syndrome	Med n fascicle × 1 to biceps	Isolated nerve transfer for isolated nerve injury	4	4	4	14
18	8	Surgery- ortho	MCN	Axillary artery lac	Med n fascicle × 1 to brachialis	Isolated nerve transfer for isolated nerve injury. Previous MCN nerve graft had restored biceps but not brachialis	4	5	20	60
42	19	Surgery- spinal	C6	Nil	Med n fascicle × 2 to biceps	Delayed referral. Due to higher transfer failure risk, ulnar n not dissected in case required for salvage	3	4	2	12
43	15	GSW	MCN	Nil	Med n fascicle × 1 to brachialis	Isolated nerve transfer for isolated nerve injury	4	5	8	21

**Table 6:** Clinical details and operative reasoning for unplanned single fascicle transfers.

**Age**	**Delay to surgery (m)**	**MOI**	**Diagnosis**	**Other injuries**	**Surgical details**	**Reason for SFT**	**Pre-Op MRC**	**Post-Op MRC**	**Post-op KG**	**Follow Up (m)**
42	5	MBA	C5,6,7+	Clavicle, C-Spine #s	Median nerve fascicle to biceps	No redundant ulnar n fascicles	0	4	1.5	14
34	4	MBA	C5,6,7	Cardiac arrest, rib #, PTx, BKA	Median nerve fascicles × 2 to biceps	No redundant ulnar n fascicles	0	4	1	80
25	9	NA	MCN	NA	Median nerve fascicle to brachialis	Small caliber ulnar n with unusual interfascicular branching, deemed not suitable for fascicle transfer	4	5	10	4
22	4	NA	Posterior cord + MCN	NA	Median nerve fascicle to biceps Subsequent nerve graft from lateral pectoral nerve to MCN	Ulnar nerve not suitable- neuroma in continuity found intra-operatively	0	4	NA	21
20	9	MBA	C5,6,7	Rib #, Brachial artery lac	Ulnar nerve fascicle to biceps	No redundant median n fascicles	0	4	7	24

The PSFT-Aug group had some elbow flexion (mostly “weak” M4, one patient M3), and the operative intent here was to augment function. The PSFT-M0 group had no elbow flexion pre-operatively, and had a single fascicular transfer planned because of preoperative assessment predicting insufficient donor fascicles to safely undergo a double fascicle transfer. Those undergoing PSFT for augmentation all had improved MRC grade and power post-operatively, whereas only one out of three patients undergoing PSFT because of insufficient donors (PSFT-M0) was able to achieve a post-operative MRC grade of >M2 (Table 5).

Previous research has described results of single nerve transfers, double nerve transfers, or rarely both, in a comparison. This study reports detailed results from a single surgeon consecutive patient cohort, which is larger than any published single surgeon series in the literature. We report excellent overall results, with 100% of patients in the DFT and USFT groups, and 96% of all patients achieving M4 or M5 strength.

In addition to the standard assessment of results by the Medical Research Council's grading system for muscle strength, strength was measured objectively with weights. The MRC grading system has been understandably criticized when applied to elbow flexion (29); but it is useful in comparing our results with those previously published in the literature. In some instances, the terminology “MRC 4+” was recorded to infer a strong grade M4; and whilst many of our patients fell in this category post-operatively, these are included in our results simply as M4, in line with the original MRC description. The quantifiable measure of kilograms lifted provides a more accurate and meaningful assessment of power, is easily reproducible, and minimizes the risk of operator bias. Whilst this outcome measure is not available for some of the more historical patients in this cohort, it has become standard in our institution to measure this pre-and post-operatively.

Our study demonstrates that pre-operative clinical assessment can predict elbow flexion outcome. Those patients who, after clinical and radiological assessment, were planned for a double nerve transfer achieved successful outcomes regardless of whether or not they ultimately underwent a single or double nerve transfer. All patients achieved M4 or better. Those who ultimately underwent double nerve transfer achieved an average elbow flexion of 6.5 kg, and those who ultimately underwent USFT achieved average elbow flexion of 4.3 kg.

The patients who underwent planned single nerve transfers fall into two groups. The PSFT-Aug group had all four patients start with M3 or M4 pre-operatively, and two of these were able to improve to M5 post-operatively, with a median lift of 6.0 kg. In stark contrast, the PSFT-M0 group all began with M0, and only one of these three patients achieved a successful outcome (M4, 2 kg lift), with the other two patients achieving only M1 and M2 power, which we believe is a reconstructive failure.

This study highlights that results of median and/or ulnar fascicular nerve transfer surgery for elbow flexion are excellent almost all of the time, with the exception of a specific subset of patients. In those three patients undergoing a PSFT due to a paucity of donors, only one was successful. This group is completely different to those patients undergoing a PSFT for augmentation of already existing elbow flexion.

Our approach to the decision regarding PSFT now involves both pre-operative assessment and when appropriate, proceeding to operative intraneural dissection of median and ulnar nerves, and selective fascicular stimulation looking for potential donors. The senior author believes that in the “borderline case” of possible nerve transfer (PSFT-M0, for paucity of donors), a careful dissection and fascicular interrogation can safely inform the final decision regarding whether or not to proceed with nerve transfer. In light of these findings, the senior author is now willing to completely abort a potential nerve transfer and undertake an alternative reconstruction should the surgical findings suggest that the alternative reconstruction has a higher likelihood of success. Locoregional muscle transfer or free functioning muscle transfer neurotised by extraplexal nerves (such as the spinal accessory nerve) can produce consistent results (30–33) and should be considered in patients with deficient ulnar and median fascicular donors.

## Conclusion

This research has allowed the authors to more accurately prognosticate and thus plan surgeries, as well as educate patients pre-operatively. The vast majority of patients can be expected to have excellent outcomes from ulnar and median fascicular transfers for elbow flexion. The very small subset of patients in whom a paucity of donor nerve availability means nerve transfer surgery is less reliable can be offered alternate reconstructions. On average we now expect MRC 4 power by 9 months post-operatively with ongoing strength gains thereafter, and eventual average quantitative elbow flexion strength of 6.5 kg where DFT is performed, and 4.3 kg where USFT is undertaken.

## Data Availability Statement

All datasets presented in this study are included in the article/supplementary material.

## Ethics Statement

The studies involving human participants were reviewed and approved by Alfred Hospital Ethics Committee. Written informed consent for participation was not required for this study in accordance with the national legislation and the institutional requirements.

## Author Contributions

SF and WA: manuscript concept, data collection, analysis, writing of manuscript, and editing.

## Conflict of Interest

The authors declare that the research was conducted in the absence of any commercial or financial relationships that could be construed as a potential conflict of interest.

## References

[1] Carlsen BT Bishop AT Shin AY. Late reconstruction for brachial plexus injury. Neurosurg Clin North Am. (2009) 20:51–64, vi. 10.1016/j.nec.2008.07.021 19064179

[2] Shin AY Spinner RJ Steinmann SP Bishop AT Adult traumatic brachial plexus injuries. J Am Acad Orthop Surg. (2005) 13:382–96. 10.5435/00124635-200510000-00003 16224111

[3] Vekris MD Beris AE Lykissas MG Korompilias AV Vekris AD Soucacos PN. Restoration of elbow function in severe brachial plexus paralysis via muscle transfers. Injury. (2008) 39 (Suppl. 3):S15–22. 10.1016/j.injury.2008.06.008 18687429

[4] Brunelli GA Vigasio A Brunelli GR. Modified Steindler procedure for elbow flexion restoration. J Hand Surg Am. (1995) 20:743–6. 10.1016/S0363-5023(05)80424-1 8522739

[5] Gutowski KA Orenstein HH. Restoration of elbow flexion after brachial plexus injury: the role of nerve and muscle transfers. Plast Reconstr Surg. (2000) 106:1348–57; quiz 1358; discussion 1359. 10.1097/00006534-200011000-00020 11083569

[6] Moneim MS Omer GE. Latissimus dorsi muscle transfer for restoration of elbow flexion after brachial plexus disruption. J Hand Surg Am. (1986) 11:135–9. 10.1016/S0363-5023(86)80121-6 3944429

[7] Tung TH Mackinnon SE. Nerve transfers: indications, techniques, and outcomes. J Hand Surg Am. (2010) 35:332–41. 10.1016/j.jhsa.2009.12.002 20141906

[8] Chuang DC Epstein MD Yeh MC Wei FC. Functional restoration of elbow flexion in brachial plexus injuries: results in 167 patients (excluding obstetric brachial plexus injury). J Hand Surg Am. (1993) 18:285–91. 10.1016/0363-5023(93)90363-8 8463596

[9] Kobayashi J Mackinnon SE Watanabe O Ball DJ Hu XM Hunter DA. The effect of duration of muscle denervation on functional recovery in the rat model. Muscle Nerve. (1997) 20:858–66. 10.1002/(SICI)1097-4598(199707)20:7<858::AID-MUS10>3.0.CO;2-O 9179158

[10] Mackinnon SE Novak CB. Nerve transfers. New options for reconstruction following nerve injury. Hand Clin. (1999) 15:643–66, ix. 10563268

[11] Weber R MacKinnon S. Nerve transfers in the upper extremity. J Hand Surg Am (2004) 4:200–13. 10.1016/j.jassh.2004.06.01128058042

[12] Tung TH Mackinnon SE Brachial plexus injuries. Clin Plast Surg. (2003) 30:269–87. 10.1016/S0094-1298(02)00094-9 12737356

[13] Oberlin C Beal D Leechavengvongs S Salon A Dauge MC Sarcy JJ. Nerve transfer to biceps muscle using a part of ulnar nerve for C5-C6 avulsion of the brachial plexus: anatomical study and report of four cases. J Hand Surg Am. (1994) 19:232–7. 10.1016/0363-5023(94)90011-6 8201186

[14] Teboul F Kakkar R Ameur N Beaulieu JY Oberlin C. Transfer of fascicles from the ulnar nerve to the nerve to the biceps in the treatment of upper brachial plexus palsy. J Bone Joint Surg Am. (2004) 86:1485–90. 10.2106/00004623-200407000-00018 15252097

[15] Loy S Bhatia A Asfazadourian H Oberlin C. [Ulnar nerve fascicle transfer onto to the biceps muscle nerve in C5-C6 or C5-C6-C7 avulsions of the brachial plexus. Eighteen cases]. Ann Chir Main Memb Super. (1997) 16:275–84. 10.1016/S0753-9053(97)80040-3 9479435

[16] Leechavengvongs S Witoonchart K Uerpairojkit C Thuvasethakul P Ketmalasiri W. Nerve transfer to biceps muscle using a part of the ulnar nerve in brachial plexus injury (upper arm type): a report of 32 cases. J Hand Surg Am. (1998) 23:711–6. 10.1016/S0363-5023(98)80059-2 9708387

[17] Sungpet A Suphachatwong C Kawinwonggowit V. One-fascicle median nerve transfer to biceps muscle in C5 and C6 root avulsions of brachial plexus injury. Microsurgery. (2003) 23:10–3. 10.1002/micr.10079 12616512

[18] Liverneaux PA Diaz LC Beaulieu JY Durand S Oberlin C. Preliminary results of double nerve transfer to restore elbow flexion in upper type brachial plexus palsies. Plast Reconstr Surg. (2006) 117:915–9. 10.1097/01.prs.0000200628.15546.06 16525285

[19] Socolovsky M Martins RS Di Masi G Siqueira M. Upper brachial plexus injuries: grafts vs ulnar fascicle transfer to restore biceps muscle function. Neurosurgery. (2012) 71(2 Suppl Operative):ons 227–32. 10.1227/NEU.0b013e3182684b51 22791036

[20] Ali ZS Heuer GG Faught RW Kaneriya SH Sheikh UA Syed IS. Upper brachial plexus injury in adults: comparative effectiveness of different repair techniques. J Neurosurg. (2015) 122:195–201. 10.3171/2014.9.JNS132823 25361485

[21] Carlsen BT Kircher MF Spinner RJ Bishop AT Shin AY. Comparison of single versus double nerve transfers for elbow flexion after brachial plexus injury. Plast Reconstr Surg. (2011) 127:269–76. 10.1097/PRS.0b013e3181f95be7 20871484

[22] Mackinnon SE Novak CB Myckatyn TM Tung TH. Results of reinnervation of the biceps and brachialis muscles with a double fascicular transfer for elbow flexion. J Hand Surg Am. (2005) 30:978–85. 10.1016/j.jhsa.2005.05.014 16182054

[23] Ray WZ Pet MA Yee A Mackinnon SE. Double fascicular nerve transfer to the biceps and brachialis muscles after brachial plexus injury: clinical outcomes in a series of 29 cases. J Neurosurg. (2011) 114:1520–8. 10.3171/2011.1.JNS10810 21351836

[24] Estrella EP. Functional outcome of nerve transfers for upper-type brachial plexus injuries. J Plast Reconstr Aesthet Surg. (2011) 64:1007–13. 10.1016/j.bjps.2011.02.002 21377436

[25] Oberlin C Durand S Belheyar Z Shafi M David E Asfazadourian H. Nerve transfers in brachial plexus palsies. Chir Main. (2009) 28:1–9. 10.1016/j.main.2008.11.010 19162520

[26] Barthel PY Barbary S Breton A Apredoaei C Dap F Mansat P. [Recovery of elbow flexion in post-traumatic C5-C6 and C5-C6-C7 palsy: retrospective dual-center study comparing single and double nerve transfer]. Chir Main. (2014) 33:211–8. 10.1016/j.main.2014.02.002 24685598

[27] Donnelly MR Rezzadeh KT Vieira D Daar D Hacquebord J. Is one nerve transfer enough? A systematic review and pooled analysis comparing ulnar fascicular nerve transfer and double ulnar and median fascicular nerve transfer for restoration of elbow flexion after traumatic brachial plexus injury. Microsurgery. (2020) 40:361–9. 10.1002/micr.30536 31755577

[28] Sneiders D Bulstra LF Hundepool CA Treling WJ Hovius SER Shin AY. Outcomes of single versus double fascicular nerve transfers for restoration of elbow flexion in patients with brachial plexus injuries: a systematic review and meta-analysis. Plast Reconstr Surg. (2019) 144:155–66. 10.1097/PRS.0000000000005720 31246823

[29] MacAvoy MC Green DP. Critical reappraisal of medical research council muscle testing for elbow flexion. J Hand Surg Am. (2007) 32:149–53. 10.1016/j.jhsa.2006.10.020 17275586

[30] Cambon-Binder A Walch A Marcheix PS Belkheyar Z. Bipolar transfer of the pectoralis major muscle for restoration of elbow flexion in 29 cases. J Shoulder Elbow Surg. (2018) 27:e330–6. 10.1016/j.jse.2018.06.027 30195620

[31] Nicoson MC Franco MJ Tung TH. Donor nerve sources in free functional gracilis muscle transfer for elbow flexion in adult brachial plexus injury. Microsurgery. (2017) 37:377–82. 10.1002/micr.30120 27704606

[32] Potter SM Ferris SI. Reliability of functioning free muscle transfer and vascularized ulnar nerve grafting for elbow flexion in complete brachial plexus palsy. J Hand Surg Eur Vol. (2017) 42:693–9. 10.1177/1753193417702029 28387564

[33] Scollan JP Newman JM Shah NV Kuehn E Koehler SM. Free gracilis muscle transfers compared with nonfree muscle flaps for reanimation of elbow flexion: a meta-analysis. J Hand Microsurg. (2020) 12:37–42. 10.1055/s-0039-1697064 32280180 PMC7141899

